# Traditional Chinese Medicine QPYF as Preventive Treatment for* Clostridium difficile* Associated Diarrhea in a Mouse Model

**DOI:** 10.1155/2016/3759819

**Published:** 2016-11-24

**Authors:** Guo Ya-Nan, Wang Jun, Zhang Hao-Jun, Jia Hong-Bing, Li Ping, Liu Xin-Zhu

**Affiliations:** ^1^Department of Pediatrics, China-Japan Friendship Hospital, Beijing, China; ^2^Clinical Research Institute, China-Japan Friendship Hospital, Beijing, China; ^3^Department of Laboratory Medicine, China-Japan Friendship Hospital, Beijing, China

## Abstract

Traditional Chinese medicine QPYF has a good effect for treating antibiotic-associated diarrhea in clinical practice. The aim of this study is to test its efficacy to prevent* Clostridium difficile* associated diarrhea (CDAD) in a mouse model. C57BL/6 mice were infected with* Clostridium difficile* VPI 10463 after exposure to antimicrobial mixture. QPYF was administered from 7 days prior to* Clostridium difficile* infection to 20 days after infection, and its effect was compared with no treatment and receiving placebo. The mice were monitored for 20 days and the percent survival, disease activity index, weight loss, colon histopathology, and the levels of toxins in the feces were measured. The expressions of TNF *α*, MCP-1, NF-*κ*B p65, and phospho-NF-*κ*B p65 in the colon were presented by immunohistochemistry. The survival rate of QPYF group (93.75%) was higher than that of model control group (65%). The mice treated with QPYF had a lower weight loss and disease activity index, compared to the mice with placebo. A significantly lower level of histopathology scores, toxins in the feces, and TNF *α*, MCP-1, NF-*κ*B p65, and phospho-NF-*κ*B p65 were detected for QPYF-treated mice. Traditional Chinese medicine QPYF showed a good preventive effect for CDAD in a mouse model.

## 1. Introduction

*Clostridium difficile* associated diarrhea (CDAD) is a major form of nosocomial infectious diarrhea [1].* Clostridium difficile* bacterium is a kind of normal gut bacterium. After using antibiotics and immunosuppressive agents, the normal intestinal microbial balance is destructed, which leads to* Clostridium difficile* booming [2]. Abundant toxins A/B are secreted, which can activate the NF-*κ*B signal pathway in monocytes and colonic epithelial cells [3]. The activation of NF-*κ*B leads to release a large number of proinflammatory factors, such as TNF *α* and MCP-1, which are the major case of intestinal inflammation [4].

Metronidazole and vancomycin are the first-line agents in treating CDAD. They have been used for 25 years. Approximately 25% of cases develop recurrent disease [5, 6]. Probiotics and vaccines are suggested to intervene CDAD, but their role is limited to the prevention [7]. In addition, feces transplantation for treating CDAD is still in the stage of research and has some problems, such as the selection of patients and donors, the preparation method, and the forward effectivity and security [8]. Thus, a new kind of method to treat CDAD is required urgently.

Traditional Chinese medicine showed a good curative effect for the diarrhea due to antibiotics. Some traditional Chinese medicines, such as rhodiola rosea, poria cocos, and codonopsis pilosula, can not only promote the growth and reproduction of beneficial bacteria, but also regulate intestinal immunity and inhibit inflammatory response [9, 10]. Recent studies have found that Chinese medicine berberine can treat CDAD by inhibiting* Clostridium difficile* bacteria and regulating the intestinal flora [11]. In vitro experiments confirmed that some TCM compound prescription can inhibit the growth of* Clostridium difficile* bacteria and the expression of toxins [12, 13]. Traditional Chinese medicine (TCM) showed a great prospect for treating CDAD. According to TCM theory, we created a method of Arousing Spleen and Tonifying Lung (QPYF) to treat CDAD. This TCM compound prescription has obtained a good effect for treating antibiotic-associated diarrhea clinically. Thus, the aim of our study is to investigate the efficacy of QPYF to prevent CDAD in a mouse model.

## 2. Materials and Methods

### 2.1. Regents

The TCM compound prescription, QPYF, was produced through water extraction form after decoction by pharmaceutical department, China-Japan Friendship Hospital (Beijing China), which consisted of rhodiola rosea, poria cocos, codonopsis pilosula, atractylodes macrocephala koidz, puerarin, rhizoma zingiberis, and liquorice. Antimicrobial agents were purchased from Sigma-Aldrich.* Clostridium difficile* strain VPI10463 (ATCC43255) was obtained from Clinical Laboratory, Beijing Friendship Hospital. Antibodies against TNF *α* and MCP-1 were purchased from Abcam (Cambridge, UK), and NF-*κ*B p65 and phospho-NF-*κ*B p65 were from Santa Cruz Biotechnology (Santa Cruz, CA, USA).

### 2.2. Animals

Nine-week-old female C57BL/6 mice were obtained from Beijing HFK Bio-technology Co., LTD. The mice were housed in groups 4–6 in individually ventilated cages at the Animal Centre of the Beijing University of Chinese Medicine. Feed and water were accessible ad libitum throughout the experiment. Upon arrival, the mice were acclimated for two weeks in the research facilities.

### 2.3. Mouse Model of CDAD

The mouse model of CDAD was induced based on the protocol described by Chen et al. [14]. An antibiotic mixture of kanamycin (0.4 mg/mL), gentamicin (0.035 mg/mL), colistin (850 U/mL), metronidazole (0.215 mg/mL), and vancomycin (0.045 mg/mL) was administered in the drinking water for 3 days. Then, the mice were switched back to regular drinking water for 2 days and received a single dose of clindamycin (10 mg/kg) intraperitoneally on day 1 prior to* Clostridium difficile* challenge. After 24 h, animals were infected with* Clostridium difficile* VPI 10463 approximately 2 × 10^4^ CFU by oral gavage. During the experiment, the mice were monitored daily for signs of disease, such as diarrhea, weight loss, and blood in feces.

### 2.4. Experiment Treatment

**Figure 1:**
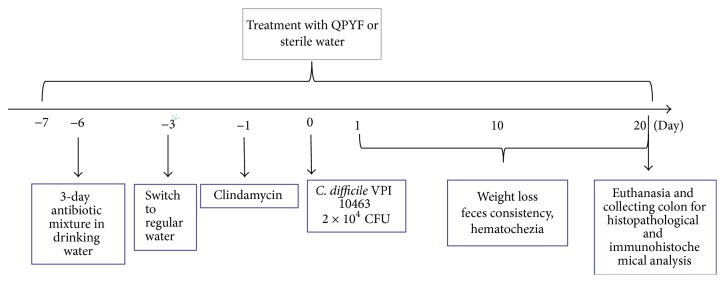
Experimental scheme.

The experimental scheme is illustrated in Figure 1. 50 mice were randomly divided into 3 groups: normal control group (*n* = 14), model control group (*n* = 20) treated with sterile water, and QPYF group (*n* = 16) treated with QPYF (4.56 g/kg/day). All treatments were administered by oral gavage from 7 days prior to* Clostridium difficile* infection to 20 days after infection. Disease severity score was shown as disease activity index (DAI), which was calculated by the following formula: percent weight loss score (0–3) + stool consistency score (0–3) + hematochezia level score (0–3) [15]. The relative weight was presented as the percentage of the original weight on day 0.

### 2.5. Histopathological Analysis

At day 20, the surviving animals were sacrificed by cervical dislocation. The colon tissue samples were collected and fixed in neutral 4% buffered formalin. Samples embedded by paraffin were sliced in 5 *μ*m sections and stained with HE for light microscopic examination. The histological analysis was graded using a scoring system reported by Chen et al. [14]. The histological scores were determined as epithelial damage (score of 0–3), neutrophil infiltration (score of 0–3), and congestion/edema (score of 0–3). An experienced histopathologist who was blinded to the group evaluated and graded all the sections.

### 2.6. *Clostridium difficile* Toxin Assay

The feces on day 4 were collected to determine the level of* Clostridium difficile* toxins.* Clostridium difficile* toxins A and B were detected by a* Clostridium difficile* toxin A and B kit (bioMérieux, Inc., France) and a Vidas immunoassay system. The assay automatically detected the quantification of* Clostridium difficile* toxins A and B in 200 mg of fecal samples using the enzyme-linked fluorescence assay (ELFA) technique. The test value for each sample can be calculated by the Vidas instrument as follows: test value = patient relative fluorescence value (RVF)/standard RVF.

### 2.7. Immunohistochemistry for TNF *α*, MCP-1, NF-*κ*B p65, and Phospho-NF-*κ*B p65

Immunohistochemistry was performed in colon tissue as previously reported. The sections embedded by paraffin were deparaffinized and rehydrated and then treated for antigen retrieval with 0.05% trypsin before blocking with 10% BSA for 1 h. Thereafter, sections were incubated overnight (4°C) with primary antibodies (TNF *α*, MCP-1, NF-*Κ*B p65, and phospho-p65) diluted 1 : 100. After being washed, the slides were incubated with biotinylated goat anti-mice IgG (Beijing ZSGB Bio Origene, “Your Gene Company”) for 30 min at 37°C. Then, the slides were visualized with the peroxidase substrate DAB. Finally, slides were counterstained with hematoxylin. 10 visions were selected randomly from each slide and mean positive area was calculated and analyzed by* Image Pro Plus*.

### 2.8. Statistical Analysis

Data analysis was performed by SPSS 16.0 statistical software. Results are expressed as mean ± standard error. The survival rates between treatment groups were analyzed by Kaplan-Meier survival analysis and compared with the log-rank test. The nonparametric data were analyzed for statistical significance using Kruskal-Wallis test and Dunns' posttest. A *P* value of <0.05 was considered to be statistically significant.

## 3. Results

### 3.1. Survival Rate after the Challenge of* Clostridium difficile*

**Figure 2:**
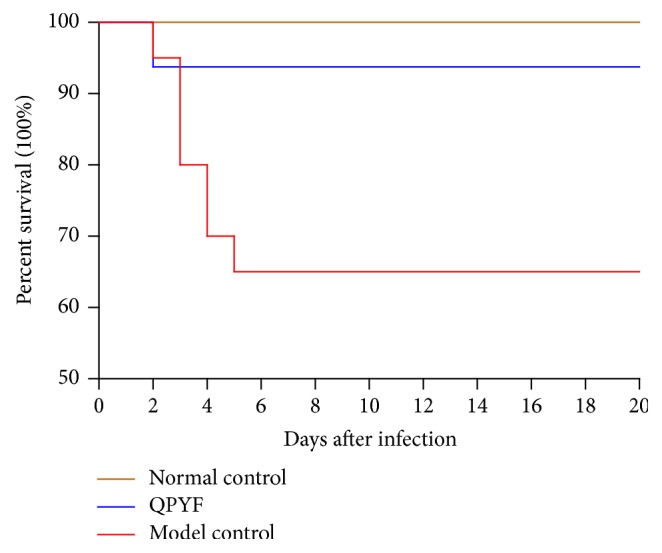
Kaplan-Meier survival plots for the Normal control group, QPYF group, and model control group after* Clostridium difficile* challenge. The survival rate of normal control group, QPYF group, and model control group was 100% (14/14), 93.75% (15/16), and 65% (13/20). The survival rate in the QPYF group was significantly higher than the model control group (the log-rank test, *P* = 0.013).

As shown in Figure 2, the infected mice died from days 2 to 5 after* Clostridium difficile* challenge. During the experiment, the survival rate of normal control group, QPYF group, and model control group was 100% (14/14), 93.75% (15/16), and 65% (13/20). We found a significantly higher survival in the QPYF group than model control group (the log-rank test, *P* = 0.013).

### 3.2. Weight Loss and Disease Severity Score

**Figure 3:**
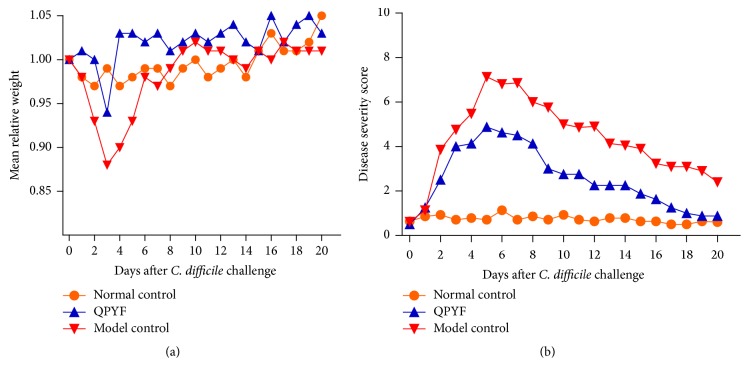
Mean relative weight (a) and disease severity score (b) were presented from days 0 to 20. Mean relative weight was the percentage of the original weight on day 0. Disease severity score was based on weight loss, the consistency of the feces, and hematochezia. On day 3 after infection, the clinical symptoms with weight loss, diarrhea, and blood in feces were the most serious. The mean relative weight of normal control group, QPYF group, and model control group were 0.99 ± 0.021, 0.94 ± 0.041, and 0.88 ± 0.045. Compared with model control group, the weight loss in QPYF group was obviously lower (*P* < 0.01). The mean disease severity score (DAI) was as follows: normal control group, 0.73 ± 0.033; model control mice, 4.28 ± 0.385; QPYF group, 2.53 ± 0.301. The mice in QPYF group had a lighter clinical symptoms than the model control mice (*P* < 0.01).

After* Clostridium difficile* challenge, the infected mice showed signs of clinical symptoms with weight loss, diarrhea, and blood in feces.  Figure 3 illustrates the mean relative weight and disease severity score among the three groups. The body weight of infected mice obviously decreased at days 2–4 after* Clostridium difficile* challenge. The weight loss was the most serious on day 3. On day 8 after* Clostridium difficile* infection, the mean weights of three groups were gradually similar. Compared to the normal control group, the mean relative weight of model control group had significant loss (normal control group, 0.99 ± 0.021 versus model control group, 0.88 ± 0.045, *P* < 0.01). Besides, the mean weight loss of QPYF group was obviously lower than the model control group (QPYF group: 0.94 ± 0.041 versus model control group: 0.88 ± 0.045  *P* < 0.01). Disease severity scores (DAI) peaked on day 5. Over time, the DAI of infected mice were close to normal group. The mean DAI was as follows: normal control group, 0.73 ± 0.033; model control mice, 4.28 ± 0.385; QPYF group, 2.53 ± 0.301. QPYF group had a lower DAI score than model control group (*P* < 0.01). So, QPYF can improve the clinical symptoms of* Clostridium difficile* bacteria infection in mice.

### 3.3. Colonic Histopathology

**Figure 4:**
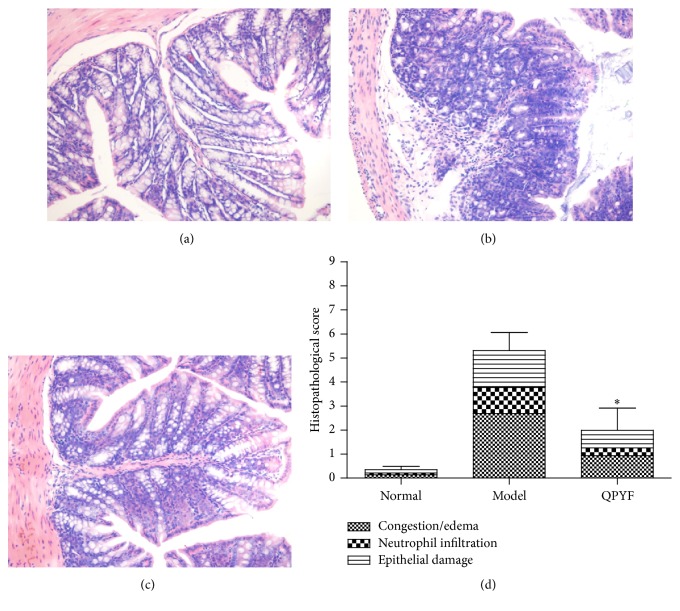
Colonic pathology. H&E-stained colon tissues of mice are shown (×200). (a) Normal control group (histopathological score: 0.35 ± 0.14) and (b) model control group (histopathological score: 5.31 ± 0.75) illustrate epithelial damage, neutrophil infiltration, and congestion/edema, (c) QPYF group (histopathological score: 1.99 ± 0.92); the degree of colon damage was less prominent than (b). (d) The histopathological score: the mouse treated with QPYF had a significantly lower score than model control group (^*∗*^ *P* < 0.01).

We analyzed the histopathological changes in colonic sites of mice receiving the different treatments to confirm the effect of QPYF (Figure 4).  Figure 4(a) showed the normal colon tissue pathology, and the histopathological score was 0.35 ± 0.14. The mice of model control group presented severe epithelial damage, neutrophil infiltration, and congestion/edema and the average histopathological score was 5.31 ± 0.75 (Figure 4(b)). For QPYF-treated mice (Figure 4(c)), the degree of colon injury was weaker than model control group (1.99 ± 0.92, *P* < 0.01). Clearly, the QPYF was effective on the improvement of colonic histopathology in the mouse model (Figure 4(d)).

### 3.4. *Clostridium difficile* Toxins in the Fecal Samples

**Figure 5:**
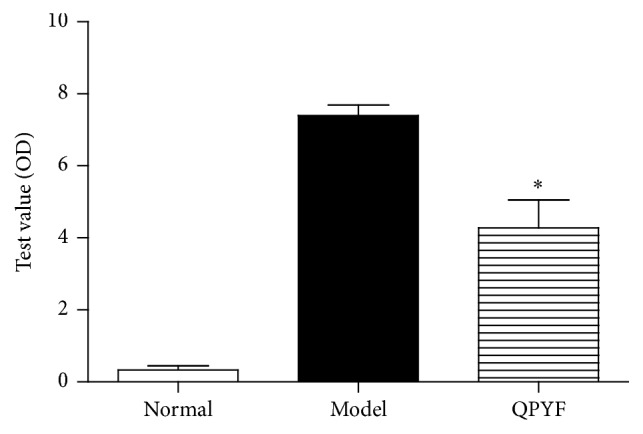
*Clostridium difficile* toxin A/B levels were measured in the fecal samples on day 4 and expressed as test value (OD). The OD values of model control group and normal control group reached 0.33 ± 0.12 and 7.40 ± 0.29. Lower mean toxin level in QPYF-treated mice (4.28 ± 0.78) was detected compared to model control group (^*∗*^ *P* < 0.01).

*Clostridium difficile* toxins in the fecal samples from mice were illustrated in Figure 5. After 4 days after* Clostridium difficile* challenge, the fecal samples were collected to detect toxins A and B. The OD value of normal control group was 0.33 ± 0.12. Toxin level of QPYF group (4.28 ± 0.78) was significantly lower than model control group (7.40 ± 0.29, *P* < 0.01). Thus, QPYF could reduce toxins A and B of mice infected* Clostridium difficile*.

### 3.5. The Expression of TNF *α*, MCP-1, NF-*κ*B p65, and Phospho-NF-*κ*B p65 in Colon Tissue

**Figure 6:**
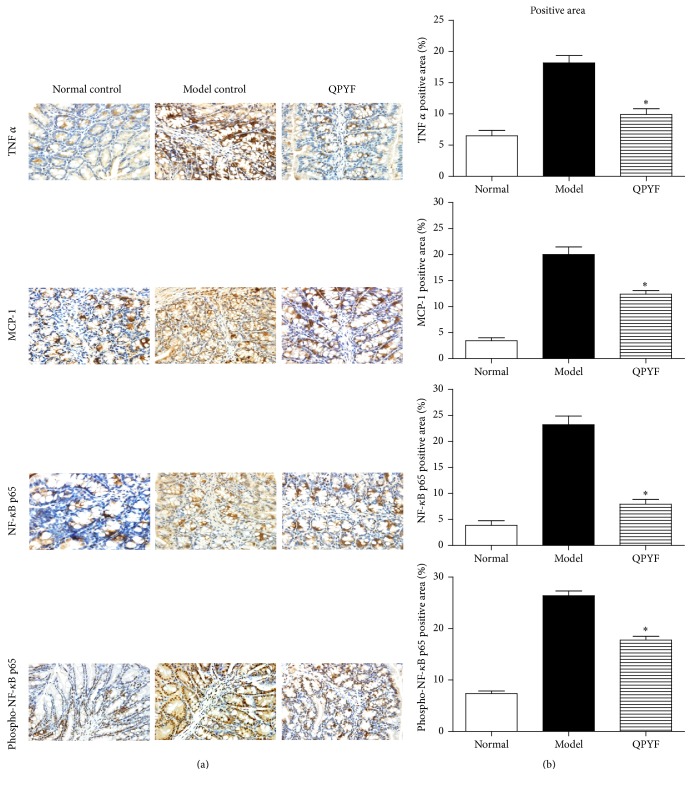
The immunohistochemical analysis of TNF *α*, MCP-1, NF-*κ*B p65, and phospho-NF-*κ*B p65 in the colon tissues of mice (×400). (a) The expression of TNF *α*, MCP-1, NF-*κ*B p65, and phospho-NF-*κ*B p65 in QPYF group was obviously weaker than the model control group. (b) The percent positive area was illustrated by* image plus pro 6.0*, and the mouse treated with QPYF had a significantly lower positive area than the model control group (^*∗*^ *P* < 0.01).

We performed immunohistochemistry in the colon tissue to detect the efficacy of QPYF on the NF-*κ*B pathway. The expression of TNF *α*, MCP-1, NF-*κ*B p65, and phospho-NF-*κ*B p65 was illustrated in Figure 6. The percent positive area was calculated by* image plus pro 6.0*. As showed in Figure 6(a), the levels of TNF *α*, MCP-1, NF-*κ*B p65, and phospho-NF-*κ*B p65 in QPYF group were obviously lower than model control group. Compared with model control group, the QPYF-treated mice had a lower percent positive area of TNF *α* (9.90 ± 2.32 versus 18.18 ± 2.82, *P* < 0.01), MCP-1 (12.39 ± 1.79 versus 20.00 ± 3.84, *P* < 0.01), NF-*κ*B p65 (7.94 ± 2.40 versus 23.21 ± 4.40, *P* < 0.01), and phospho-NF-*κ*B p65 (17.77 ± 2.18 versus 26.30 ± 2.78, *P* < 0.01, Figure 6(b)).

## 4. Discussion

With the wide application of broad-spectrum antibiotics, the incidence of CDAD is gradually rising. The search for alternative treatments against CDAD is becoming more and more urgent [16]. TCM has a history of thousands of years, and it has its own unique theory on CDAD. After our previous studies, most doctors of TCM think the internal cause of CDAD is intestinal dysbiosis and the external cause is intrusion of* Clostridium difficile*. Antibiotics belong to bitter and cold objects, which easily damage Yang spirit of the body. Long-term use of antibiotics can waste Spleen Yang and Kidney Yang. Due to the insufficient Yang spirit, the transportation and transformation function of Spleen are abnormal, which leads to wetness production in Middle Jiao. Then, abdominal distension, abdominal pain, and diarrhea occur, because the QI depression is retarded by wetness. If wetness stands for a long time, it can be converted into heat and poison, causing fever and hematochezia [17].

According to the theory, we set up a method of Arousing Spleen and Tonifying Lung (QPYF) to treat CDAD. The aim is to clear wetness by recovering the function of Spleen by Arousing Spleen and to evacuate water fluid and improve immunologic function by Tonifying Lung. The compound prescription of QPYF consisted of rhodiola rosea, poria cocos, codonopsis pilosula, atractylodes macrocephala koidz, puerarin, rhizoma zingiberis, and liquorice. The recipe is not only clearing wetness by Arousing Spleen but also improving immunologic function by Tonifying Lung. It has been used clinically and obtained a good effect.

This murine model of CDAD described by Fitzpatrick et al. was widely used for testing the efficacy of applicable pharmacological agents [18]. We successfully copied out CDAD model according to the method. After* Clostridium difficile* infection, the mice exhibited clinical symptoms, such as weight loss, diarrhea, and even death. Treatment results of three groups indicate that the mortality of mice given QPYF treatment in advance was obviously lower than model control group (Figure 2). Compared with the infected mice treated with sterile water, the clinical symptoms of the QPYF-treated mice, such as weight loss, diarrhea, and hematochezia, were much lighter and easier to restore (Figure 3). The degree of colonic damage in QPYF group was also evidently lighter than the model control group (Figure 4). In addition, we detected the toxins A/B in the feces at day 4 after* Clostridium difficile* challenge and we found that the quantity of toxins A/B in QPYF group was lower than the model control group coincidently (Figure 5).

In addition, the activation of NF-*κ*B signal pathway in monocytes and colonic epithelial cells was closely associated with the intestinal inflammation due to* Clostridium difficile* [19, 20]. Previous studies reported that toxin A secreted by* Clostridium difficile* can activate the pathway and a large number of proinflammatory factors are secreted to lead to colon inflammation [21]. We detected the expression of TNF *α*, MCP1, NF-*κ*Bp65, and phospho-NF-*κ*Bp65 in colon tissue by immunohistochemistry and found that QPYF can reduce the expression of TNF *α*, MCP1, NF-*κ*B p65, and phospho-NF-*κ*B p65 (Figure 6). QPYF had an impact on inhibiting the NF-*κ*B pathway activation and reducing the release of proinflammatory factor. Thus, QPYF given in advance can be a kind of new method on treatment of CDAD.

As far as we know, there have been no documents reported to treat CDAD by using TCM compound prescription. From the results of our study, TCM compound prescription, QPYF, given in advance has a good effect on treating CDAD. Although the mechanism of the prescription is not yet clear in modern medicine, it may provide a new approach to prevent and treat CDAD in clinical practice. The limitations of this study include the experimental design. There should have been another group that received vancomycin as positive control group in order to compare the efficiencies between QPYF and vancomycin. Nevertheless, we can still find its efficiency to prevent CDAD.

In conclusion, our results suggest that TCM compound prescription QPYF given in advance can reduce mortality, improve the clinical symptoms, relieve the degree of colonic damage, reduce the secretion of toxins, and inhibit the NF-*κ*B pathway activation for CDAD mice. The method of QPYF has a good preventive effect on treatment of CDAD.
